# Whole-genome sequence of *Limnospira fusiformis* NRMCF6962, isolated from lake water

**DOI:** 10.1128/mra.00855-24

**Published:** 2024-10-14

**Authors:** Fayaazuddin Thajuddin, Chandru Ashok, Shakena Fathima Thajuddin, Thajuddin Nooruddin, Dhanasekaran Dharumadurai, Olubukola Oluranti Babalola

**Affiliations:** 1 National Repository for Microalgae and Cyanobacteria – Freshwater (NRMC-F) (Sponsored by the DBT, Govt. of India), Bharathidasan University, Tiruchirappalli, Tamil Nadu, India; 2 Department of Microbiology, Bharathidasan University, Tiruchirappalli, Tamil Nadu, India; 3 Department of Microbiology, Jamal Mohamed College, Tiruchirappalli, Tamil Nadu, India; 4 Food Security and Safety Focus Area, Faculty of Natural and Agricultural Sciences, North-West University, Mmabatho, South Africa; Montana State University, Bozeman, Montana, USA

**Keywords:** cyanobacteria, whole-genome sequencing, nutraceutical, bioinformatics

## Abstract

The economically important Cyanobacterial genus *Limnospira* is commercially utilized as a dietary supplement and nutraceutical agent. We present the whole-genome sequence of *Limnospira fusiformis* NRMCF6962 and was isolated from Kondakarla Ava Lake of Visakhapatnam, Andhra Pradesh, India.

## ANNOUNCEMENT

*Limnospira* is an entirely novel genera in Cyanobacteria which encompasses three species with commercial significance which were previously classified under *Arthrospira* (1). The strains of *Arthrospira* and *Limnospira* are widely known for their diverse applications as food & feed additives (2–4). As a part of our investigations on identifying the regulation of genes in the Cyanobacterial genome, the isolate—NRMCF 6962 was collected from Kondakarla Ava Lake, an alkaline (pH 8.9) environment in Visakhapatnam, Andhra Pradesh (17°36′07.0″ N, 82°59′45.2″ E). Samples of water were collected from the lake during November 2023 over 0.5 m of average depth and were proceeded for filtration by plankton net with the mesh size of 15 µm, loaded with the conventional Zarrouk medium (5). The identified taxa were purified by streak plate method and inoculated in Zarrouk broth for Cyanobacterial growth under fluorescence white lamps (50 µmol photons/m²/s) at 12 h. (light/day) 25 ± 2°C for 30 days (5). DNA was extracted from auxenic culture of *L. fusiformis via* Xploregen discoveries bacterial gDNA extraction kit and quantified with a Qubit 3 fluorometer. Qubit double-stranded DNA (dsDNA) high sensitivity (HS) assay kit was employed to measure DNA using the Qubit 3 fluorometer. 100 ng of DNA was fragmented into 200–300 base pair fragments using the KAPA method (6). A-tailing enzyme mix and HyperPlus end repair are utilized on the fragmented samples for addition of A-tailing and end repair. DNA fragments were attached with adapters through ligation and the resulting library was allowed for AMPure bead purification. Qiagen NEBNext Ultra II DNA library kit was used to generate a sequencing library, which was subsequently amplified by applying NEBNext Ultra II Q5 Master Mix according to the manufacturer’s procedure. The DNA library was extracted in 15 µL of 0.1× Tris-EDTA buffer and measured with a dsDNA HS kit. The Biokart India Pvt. Ltd. in Bangalore utilized an Agilent DNA 7500 chip for fragment analysis. DNA sequencing was done using the Illumina HiSeq 4000 machine. The readings of the DNA sequence with an average length of 151 bp were allowed for quality checking with the help of FastQC v0.11.2 and were filtered by Trim Galore v0.6.4 (7, 8). Quality trimmed data were assembled using Unicycler v0.48 tool (9). By applying the gathered data along with the multilocus sequence typing (MLST) approach in PubMLST (https://pubmlst.org/) (10), species were verified and an appropriate reference sequence was acquired from National Centre for Biotechnology Information (NCBI). CONTIGuator software was used to align the resulting fasta files, containing n number of contigs (sequences), with reference genome *L. fusiformis* NRMCF6962 (https://www.ncbi.nlm.nih.gov/taxonomy/3156243) (11).

We documented a whole-genomic sequence for *L. fusiformis* NRMCF6962, with a total of 6.8 M Illumina reads and 189.36× genome coverage contained 5,534,152 bp with a GC content of 45.8%. Prokaryotic Genome Annotation Pipeline (PGAP) v5.3 revealed a total of 5,177 coding sequences (CDS) (12), including 4,993 protein-coding genes and 48 RNA-coding genes, 440 contigs, 4 CRISPR repeat units, and *N50* and L50 values of 31,828 bp and 56, respectively.

## Data Availability

This whole-genome project and associated data have been deposited in the NCBI database under the accession number JBFUXN010000000, with BioProject accession number PRJNA1115962, BioSample accession number SAMN41518288, and SRA accession number SRS21437750.
